# Synthesis and Antiplatelet Activity Evaluation of a Group of Novel Ethyl Acetoacetate Phenylhydrazone Derivatives

**DOI:** 10.22037/ijpr.2020.114123.14674

**Published:** 2021

**Authors:** Sarveen Farhady, Farzad Kobarfard, Lotfollah Saghaei, Mahboubeh Rostami

**Affiliations:** a *Department of Medicinal Chemistry, School of Pharmacy and Pharmaceutical Sciences, Isfahan University of Medical Sciences, Isfahan, Iran. *; b *Department of Medicinal Chemistry, School of Pharmacy, Shahid Beheshti University of Medical Sciences, Tehran, Iran.*

**Keywords:** Anti-platelet, Phenylhydrazone, Ethyl acetoacetate (EAA), Arachidonic acid (AA), Adenosine diphosphate (ADP)

## Abstract

A group of Novel phenylhydrazone derivatives of ethyl acetoacetate was synthesized and evaluated for their antiplatelet activities. Fourteen ethyl acetoacetate phenylhydrazone derivatives were synthesized using the diazonium salt of various aromatic primary amines with good yields and purity. The structure of the final compounds was confirmed and approved by spectroscopic techniques such as ^1^HNMR, FTIR, and ESI-Mass. We examined the antiplatelet activity of the derivatives against Arachidonic Acid (AA) and Adenosine Diphosphate (ADP) as platelet aggregation inducers. The final results indicated the acceptable potency for different derivatives. In this regard, *the para*-hydroxyphenylhydrazine derivative of ethyl acetoacetate has the best activity among all derivatives, both on AA and ADP pathways. It seems that the derivatives with electron-releasing substituents (hydroxyl, methoxy, and methyl group) have better inhibition activities against the aggregation induced by AA. In contrast, those with an electron-withdrawing group showed a significant decrease in their potency. Based on the results of this study, we would proceed with further assessments both *in-vitro* and *in-vivo* to get success in introducing some new antiplatelet agents to the clinic.

## Introduction

Cardiovascular diseases (CVDs), including coronary, cerebrovascular, and rheumatic heart diseases, are the primary causes of death worldwide. They are responsible for 17.9 million deaths (31%) in 2016, and the leading cause of these deaths is stroke and heart attack. Over 75% of CVDs deaths occur in low and middle-income countries (1).

Platelets play a crucial role in preventing blood loss in response to injury. However, they are also crucial for the formation of thrombus in atherothrombotic diseases, the principal reason for CVDs (2).

There are multiple routes in platelet activation, binding of specific agonists, such as TXA_2_ (thromboxane A_2_), ADP (adenosine diphosphate), and thrombin to their corresponding receptors on the platelet surface are some of the most reported ones (2). 

Therefore, antiplatelet drugs such as aspirin (a nonselective irreversible inhibitor of the enzyme cyclooxygenase (COX-1), which prevents the transformation of AA to its potent aggregation inducer metabolite, thromboxane A2 (TXA_2_), and clopidogrel (an irreversible antagonist of ADP) are used for the prevention and management of thrombotic disorders (3).

However, despite the advantages of antiplatelet agents, they have essential clinical limitations, including high residual risk for ischemic events, bleeding risk, headache, gastric erosion, and variable inhibition of platelet aggregation (3, 4). Therefore, efforts to find newer therapies and agents to coach these limitations and improve efficacy are a serious demand.

Based on the literature, hydrazone moiety plays a pharmacophoric role in compounds with antiplatelet aggregation activities. Todeschini *et al*. reported a group of pyridylhydrazones with an inhibitory effect against AA (5). Chelluci *et al*. reported the drugs hybridized with N-acyl hydrazone showed antiplatelet activity (6). Moreover, Barreiro *et al*. synthesized 10H-phenothiazine-1-acyl hydrazone derivatives that inhibit the AA pathway (7). N-substituted-phenyl-1,2,3-triazole-4-acyl hydrazine (8) and arylsulfonate-acyl hydrazine (9) are other examples of hydrazone compounds with antiplatelet aggregation activity.

Furthermore, the indole hydrazone derivatives were reported to have efficient platelet aggregation inhibition activity (10-14). Mashayekhi *et al*. reported a novel indole-hydrazone with an inhibitory effect against platelet aggregation induced by AA in comparison to indomethacin (11). In other studies, Tehrani *et al.* discovered a series of novel Schiff bases derived from 2-Hydrazinyl-1,3,4-thiadiazole with antiplatelet activities. Their derivatives effectively inhibited platelet aggregation at 100 µM concentration, and some of them exhibited activities comparable to those of aspirin and indomethacin. They claimed that any changes in the delocalization pattern of electrons on the structure lead to a significant drop in potency (12). Based on other studies, the electron-rich heterocyclic aldehyde hydrazones (furan, thiophene, and indole) exhibit more potency as antiplatelets (13). Furthermore, an increase in lipophilic identity is accompanied by a reduction in potency, and compounds with lower molecular weights can be considered as the lead compounds, preferably (12, 13).

**Figure 1:**
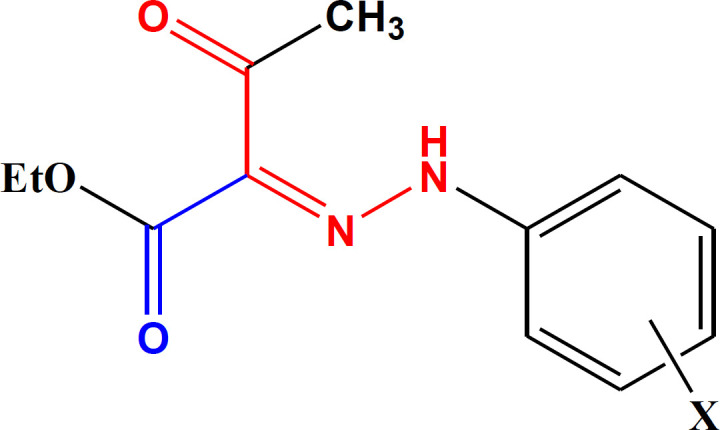
The general structure of the synthesized derivatives (3a-n).

In an attempt to find some structures with potent antiplatelet activities, based on the studies mentioned above, we synthesized aryl hydrazone derivatives of ethyl acetoacetate (Figure 1). We aimed to evaluate the effects of simultaneous conjugation of the hydrazone group with the ketone and ester moieties on the induced antiplatelet activities.

## Experimental

*General Methods*

All chemicals and solvents were purchased from Merck. ^1^HNMR spectra were recorded on a 400 MHz Bruker spectrometer in CDCl_3_ and tetramethylsilane (TMS) was used as an internal standard. ESI-MS mass spectra were recorded on an Agilent 6410 triple quadrupole mass spectrometer. IR spectra were recorded on a PerkinElmer IR spectrophotometer as potassium bromide discs. Melting points of the compounds were measured on a 9100 Electrothermal melting point apparatus.

*General Procedure for the Synthesis of Hydrazone derivatives (3a–n)*

To a solution of appropriate aniline (9.8 mmol) and 37% hydrochloric acid (2.5 mL) in water (1.5 mL) and ethanol 96% (1.5 mL), we added drop by drop a solution of sodium nitrite (10.63 mmol) in water (4.25 mL) at 0 °C. The reaction mixture was stirred at 0 °C for 30 min and then a solution of sodium acetate (38.5 mmol) in water (5 mL) and a solution of ethyl acetoacetate (9.8 mmol) in ethanol 96% (2.5 mL) were added while maintaining the temperature below 5 °C. The reaction mixture was stirred at a temperature below 5 °C, and the reaction progress was monitored by thin-layer chromatography (TLC). After the completion of the reaction, water was then added and the resulting precipitate was filtered and purified by recrystallization in an appropriate solvent or flash chromatography (17).

*In-vitro platelet aggregation assessments*

We took 10 mL of whole blood from healthy volunteers who had not taken any drugs, especially NSAIDs, 14 days before the test. The blood was mixed with trisodium citrate dihydrate 3.8% (one part citrate, 9-part blood), and the mixture was centrifuged at 1000 rpm for 8 min at room temperature to obtain platelet-rich plasma (PRP). The remained blood was centrifuged at 3000 rpm for 15 min, and platelet-poor plasma (PPP) was removed and then used as the blank.

Platelets were counted under the microscope, and the count was adjusted to 25000/mL. The 1 mM solution of synthesized compounds with DMSO was prepared. Then 1µL of the derivatives were added to 200 µL PRP in the test cuvettes and incubated for 5 min. Aggregation was induced by the addition of 5 µL of ADP (5 *μ*M) or AA (1.25 mg/mL), and it was measured with an aggregometer (APACT 4004) for 5 min at 37 °C. According to our previous studies, DMSO was used as a negative control, and indomethacin and aspirin were used as standard drugs. The extent of aggregation was quantified by determining the maximum height of the curve. Inhibition activities of the derivatives were expressed as a percentage, and the active compounds (>50% inhibition) were diluted to obtain IC_50_ values. IC_50_ values were calculated by Graph-Pad Prism version 3.02. (11, 15 and 18).

*Docking studies*

The crystal structure of COX-1 (PDB ID: 3HS5) was retrieved from the RCSB Protein Data Bank (http://www.rcsb.org). The docking studies were performed with GOLD Protein-Ligand Docking Software (19). We prepared the protein by removing all water molecules and ligands; the polar hydrogens were added by Discovery Studio V3.5. The whole 3HS5 was defined as a receptor, and the site sphere was selected based on the binding site of 3HS5. All compounds were constructed using CHEMOFFICE 3D ULTRA 13.0 software, and then they were energetically minimized by using MMFF94 with 1000 cycles and a minimum RMS gradient of 0.01 by HYPERCHEM V8. Compounds were placed during the molecular docking procedure with gold score mode for searching function and 150 runs for each ligand. Types of interactions of the docked protein with ligand were analyzed after the end of molecular docking by Pymol and Discovery studio V3.5 (20).

## Results and Discussion

*Chemistry*

**Scheme 1:**
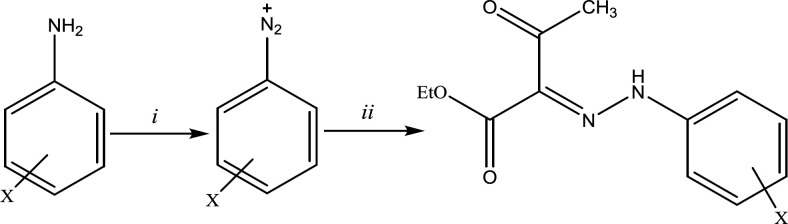
Synthetic route for the desired derivatives; reagents and conditions: (*i*) HCl 37%, ethanol, H_2_O, NaNO_2_, 0 °C. (*ii*) NaCH_3_CO_2_, ethyl acetoacetate, 0 °C

As disclosed in Scheme 1, the final hydrazones *(3a-n)* were prepared by converting aromatic amines to their diazonium salt followed by nucleophilic attack of ethyl acetoacetate carbanion to it, adding ethyl acetoacetate to the diazonium salt. The spectral data were in complete agreement with the structure of the desired derivatives. 

Spectral data for all synthesized derivatives have been provided as follow:

*Ethyl-2-(2-phenylhydrazineylidene)-3-oxobutanoate (3a)*

Yield 70%, m.p. 56.7-59.4 °C,^ 1^H NMR (400 MHz, CDCl_3_) *δ*: 14.82 (s,1H, NH), 7.41 (m, 4H, Ar C_2,3,5,6_-H), 7.18 (t, *J *= 8.0 Hz, 1H, Ar C_4_-H), 4.34 (q, *J *= 8.0 Hz, 2H, ethyl), 2.60 (s, 3H, methyl), 1.41 (t, *J *= 8.0 Hz, 3H, ethyl); IR (cm^-1^): 1723, 1527, 781, 707. ESI-MS *m/z*: 256 (M-Na^+^).

*Ethyl-2-(2-(4-chlorophenyl) hydrazineylidene)-3-oxobutanoate (3b)*

Yield 72%, m.p. 79.8-80.7 °C,^ 1^H NMR (400 MHz, CDCl_3_) *δ*: 14.76 (s,1H, NH), 7.35 (s, 4H, Ar C_2,3,5,6_-H), 4.34 (q, *J *= 8.0 Hz, 2H, ethyl), 2.59 (s, 3H, methyl), 1.40 (t, *J *= 8.0 Hz, 3H, ethyl); IR (cm^-1^): 1697, 1526, 1370, 1094, 823. ESI-MS *m/z*: 290.8 (M-Na^+^).

*Ethyl-2-(2-(4-bromophenyl) hydrazineylidene)-3-oxobutanoate (3c)*

Yield 81%, m.p. 76.2-77.3 °C,^1^HNMR (400 MHz, CDCl_3_) *δ*: 14.73 (s,1H, NH), 7.50(d, *J *= 8.0 Hz, 2H, Ar C_3,5_-H), 7.30(d, *J *= 8.0 Hz, 2H, Ar C_2,6_-H), 4.34 (q, *J *= 8.0 Hz, 2H, ethyl), 2.59 (s, 3H, methyl), 1.40 (t, *J *= 8.0 Hz, 3H, ethyl); IR (cm^-1^): 1701, 1620, 1525, 1095, 825. ESI-MS *m/z*: 336 (M-Na^+^).

*Ethyl-2-(2-(p-tolyl) hydrazineylidene)-3-oxobutanoate (3d)*

Yield 55%, m.p. 64-67.5 °C, a,b isomers, ^1^HNMR (400 MHz, CDCl_3_) *δ*: 14.90 (s,1H, NH (*b*)), 12.85 (s, 1H, NH (*a*)), 7.25 (dd, *J *= 8.4 Hz, 4H, Ar C_2,3,5,6_-H (*a,b*)), 4.32-4.40 (m, *J *= 7.2 Hz, 2H, ethyl (*a,b*)), 2.59 (s, 3H, methyl (*b*)), 2.49 (s, 3H, methyl (*a*)), 2.35 (s, 3H, Ar C_4_-CH_3_ (*a,b*)), 1.40 (t, *J *= 8.0 Hz, 3H, ethyl (*a,b*)); IR (cm^-1^): 1691, 1625, 1521, 1183, 1082, 824. ESI-MS *m/z*: 271(M-Na^+^).

*Ethyl-2-(2-(3-nitrophenyl) hydrazineylidene)-3-oxobutanoate (3e)*

Yield 82%, m.p. 118.8-119.7 °C,^1^HNMR (400 MHz, CDCl_3_) *δ*: 14.66(s,1H, NH), 8.27(s, 1H, Ar C_2_-H), 8.01 (d, *J *= 8.0 Hz, 1H, Ar C_4_-H), 7.70 (d, *J *= 8.0 Hz, 1H, Ar C_6_-H), 7.56 (t, *J *= 8.0 Hz, 1H, Ar C_5_-H), 4.37 (q, *J *= 8.0 Hz, 2H, ethyl), 2.62 (s, 3H, methyl), 1.43 (t, *J *= 8.0 Hz, 3H, ethyl); IR (cm^-1^): 1700, 1622, 1519, 1178, 881,798, 730. ESI-MS *m/z*: 280 (M-H^+^), 302 (M-Na^+^).

*Ethyl-2-(2-(2-nitrophenyl) hydrazineylidene)-3-oxobutanoate (3f)*

Yield 85%, m.p. 95.5-98.2°C,^1^HNMR (400MHz, CDCl_3_) *δ*: 13.92 (s,1H, NH), 8.27 (d, *J=*8.0 Hz, 1H, Ar C_3_-H), 8.04 (d, *J *= 8.0 Hz, 1H, Ar C_6_-H), 7.70 (t, *J *= 8.0 Hz, 1H, Ar C_5_-H), 7.20 (t, *J*=8.0 Hz, 1H, Ar C_4_-H), 4.47 (q, *J *= 8.0 Hz, 2H, ethyl), 2.55 (s, 3H, methyl), 1.43 (t, *J *= 8.0 Hz, 3H, ethyl); IR (cm^-1^): 1682, 1499, 1334, 1162, 794. ESI-MS *m/z*: 280 (M-H^+^), 302 (M-Na^+^).

*Ethyl-2-(2-(4-nitrophenyl) hydrazineylidene)-3-oxobutanoate (3g)*

Yield 51%, m.p. 116.9-118.1°C,^1^HNMR (400MHz, CDCl_3_) *δ*: 12.72 (s,1H, NH), 8.29 (d, *J*=8.8 Hz, 2H, Ar C_3,5_-H), 7.41 (d, *J *= 8.8 Hz, 2H, Ar C_2,6_-H), 4.40 (q, *J *= 7.2 Hz, 2H, ethyl), 2.53 (s, 3H, methyl), 1.41 (t, *J *= 7.2 Hz, 3H, ethyl); IR (cm^-1^): 1680, 1590, 1529, 1333, 1217, 850. ESI-MS *m/z*: 279.8 (M-H^+^), 301.8 (M-Na^+^).

*Ethyl-2-(2-(3,4-dichlorophenyl) hydrazineylidene)-3-oxobutanoate (3h)*

Yield 58%, m.p. 88.2-90.5°C, a,b isomers, ^1^HNMR (400MHz, CDCl_3_) *δ*: 14.61 (s,1H, NH (*a,b*)), 7.55 and 7.55 (each s, 1H, Ar C_2_-H (*a,b*)), 7.43 (d, *J *= 8.8 Hz, 1H, Ar C_5_-H (*a,b*)), 7.21 (d, *J=*8.8 Hz, 1H, Ar C_6_-H (*b*)), 7.20 (d, *J *= 8.8 Hz, 1H, Ar C_6_-H (*a*)), 4.35 (q, *J *= 7.2 Hz, 2H, ethyl (*a,b*)), 2.59 (s, 3H, methyl (*a,b*)), 1.40 (t, *J *= 7.2 Hz, 3H, ethyl (*a,b*)); IR (cm^-1^): 1709, 1631, 1529, 1372, 1204. ESI-MS *m/z*: 324.7 (M-Na^+^).

*Ethyl-2-(2-(3-chlorophenyl) hydrazineylidene)-3-oxobutanoate (3i)*

Yield 90%, m.p. 57.6-59.0°C, ^1^HNMR (400MHz, CDCl_3_) *δ*: 14.64 (s,1H, NH), 7.47 (s, 1H, Ar C_2_-H), 7.30 (t, *J*=8.0 Hz, 1H, Ar C_5_-H), 7.23 (d, *J *= 8.0 Hz, 1H, Ar C_6_-H), 7.13 (d, *J*=8.0 Hz, 1H, Ar C_4_-H), 4.36 (q, *J *= 7.2 Hz, 2H, ethyl), 2.59 (s, 3H, methyl), 1.41 (t, *J *= 7.2 Hz, 3H, ethyl); IR (cm^-1^): 1706, 1520, 1375, 1192, 883, 798, 684. ESI-MS *m/z*: 290.8 (M-Na^+^).

*Ethyl-2-(2-(2,6-dimethylphenyl) hydrazineylidene)-3-oxobutanoate (3j)*

Yield 45%, m.p. 53.3-56.4°C, a,b isomers, ^1^HNMR (400MHz, CDCl_3_) *δ*: 14.94 (s,1H, NH (*a,b*)), 7.03-7.09 (m, 3H, Ar C_3,4,5_-H (*a,b*)), 4.38 (q, *J *= 7.2 Hz, 2H, ethyl (*b*)), 4.28 (q, *J *= 7.2 Hz, 2H, ethyl (*a*)), 2.62 (s, 3H, methyl (*a,b*)), 2.44 and 2.43 (each s, 6H, Ar C_2,6_-CH_3_ (*a,b*)), 1.41(t, *J *= 7.2 Hz, 3H, ethyl (*b*)), 1.35 (t, *J *= 7.2 Hz, 3H, ethyl (*a*)); IR (cm^-1^): 1689, 1506, 1361, 1187, 780. ESI-MS *m/z*: 263 (M-H^+^).

*Ethyl-2-(2-(o-tolyl) hydrazineylidene)-3-oxobutanoate (3k)*

Yield 58%, m.p. 54.9-59.3°C,^1^HNMR (400MHz, CDCl_3_) *δ*: 13.04 (s,1H, NH), 7.65 (d, *J*=8.0 Hz, 1H, Ar C_3_-H), 7.28 (t, *J *= 8.0 Hz, 1H, Ar C_5_-H), 7.19 (d, *J*=8.0 Hz, 1H, Ar C_6_-H), 7.07 (t, *J*=8.0 Hz, 1H, Ar C_4_-H), 4.38 (q, *J *= 7.2 Hz, 2H, ethyl), 2.51 (s, 3H, methyl), 2.38 (s, 3H, Ar C_2_-CH_3_), 1.41 (t, *J*=7.2 Hz, 3H, ethyl); IR (cm^-1^): 1700, 1507, 1368, 1174, 1083, 764. ESI-MS *m/z*: 249 (M-H^+^), 271 (M-Na^+^).

*Ethyl-2-(2-(2,3-dimethylphenyl)hydrazineylidene)-3-oxobutanoate (3l)*

Yield 57%, m.p. °C, a,b isomers, ^1^HNMR (400MHz, CDCl_3_) *δ*: 15.17 (s,1H, NH (*b*)), 13.14 (s, 1H, NH (*a*)), 7.68 (d, *J *= 8.0 Hz, 1H, Ar C_6_-H (*b*)), 7.54 (d, *J *= 8.0 Hz, 1H, Ar C_6_-H (*a*)), 7.18 (t, *J *= 8.0 Hz, 1H, Ar C_4_-H (*a,b*)), 7.00 (t, *J *= 8.0 Hz, 1H, Ar C_5_-H (*a,b*)), 4.39 (q, *J *= 7.2 Hz, 2H, ethyl (*b*)), 4.34 (q, *J*=7.2 Hz, 2H, ethyl (*a*)), 2.62 (s, 3H, methyl (*b*)), 2.51 (s, 3H, methyl (*a*)), 2.33 (d, *J *= 4 Hz, 3H, Ar C_2_-CH_3 _(*a,b*)), 2.28 (s, 3H, Ar C_3_-CH_3_ (*a,b*)), 1.40 (dt, *J *= 7.2 Hz, *J *= 2.4 Hz, 3H, ethyl (*a,b*)); IR (cm^-1^): 1692, 1592, 1517, 1366, 1202, 1084. ESI-MS *m/z*: 263 (M-H^+^), 285 (M-Na^+^).

*Ethyl-2-(2-(4-hydroxyphenyl) hydrazineylidene)-3-oxobutanoate (3m)*

Yield 80%, m.p. 163-166.5°C, a,b isomers, ^1^HNMR (400MHz, CDCl_3_) *δ*: 15.07(s, 1H, NH (*b*)), 13.00 (s, 1H, NH (*a*)), 7.33 (d, *J *= 8.8 Hz, 2H, Ar C_2,6_-H (*b*)), 7.25 (d, *J *= 8.8 Hz, 1H, Ar C_2,6_-H (*a*)), 6.88 (dd, *J *= 8.8 Hz, *J *= 4.4 Hz, 2H, Ar C_3,5_-H (*a,b*)), 4.35 (dq, *J*=7.2 Hz, *J *= 4.4 Hz, 2H, ethyl (*a,b*)), 2.58 (s, 3H, methyl (*b*)), 2.49 (s, 3H, methyl (*a*)), 1.40 (dt, *J *= 7.2 Hz, *J *= 4.0 Hz, 3H, ethyl (*a,b*)); IR (cm^-1^): 3134, 1659, 1592, 1517, 1373, 1220, 830. ESI-MS *m/z*: 249 (M - H^+^).

*Ethyl-2-(2-(4-methoxyphenyl) hydrazineylidene)-3-oxobutanoate (3n)*

Yield 70%, m.p. 60-63.4°C, a,b isomers, ^1^HNMR (400MHz, CDCl_3_) *δ*: 15.07 (s, 1H, NH (*b*)), 12.95 (s, 1H, NH (*a*)), 7.37 (d, *J *= 8.8 Hz, 2H, Ar C_2,6_-H (*b*)), 7.29 (d, *J *= 8.8 Hz, 1H, Ar C_2,6_-H (*a*)), 6.93 (dd, *J *= 8.8 Hz, *J *= 4.0 Hz, 2H, Ar C_3,5_-H (*a,b*)), 4.34 (dq, *J *= 7.2 Hz, *J *= 5.2 Hz, 2H, ethyl (*a,b*)), 3.82 (s, 3H, Ar C_4_-OCH_3_ (*a,b*)) 2.58 (s, 3H, methyl (*b*)), 2.48 (s, 3H, methyl (*a*)), 1.40 (t, *J*=7.2 Hz, 3H, ethyl (*a,b*)); IR (cm^-1^): 1700, 1609, 1515, 1368, 1186, 834. ESI-MS *m/z*: 263 (M - H^+^).

According to the ^1^HNMR spectra of the Compounds *3d, 3h, 3l, 3m, 3n,* the duplicate nature of some peaks was observed, which could be assigned to their isomerization. In the ^1^H-NMR spectra, the hydrogen of the NH group appeared at 12.00-15.00 ppm as a singlet. A set of a quartet at ~ 4.30 ppm, a singlet at ~ 2.50 ppm, and a triplet at ~ 1.40 ppm were characteristic peaks for EAA (ethyl 3-oxobutanoate). In IR spectra, the N-H stretch band appeared weak or vanishing in the range of 3300-3500 cm^-1^(10). The molecular mass of the synthesized compounds was analyzed by ESI-MS; the molecular ions of the compounds were observed as adducts of hydrogen and/or sodium.

*Antiplatelet Activity*

**Figure 2:**
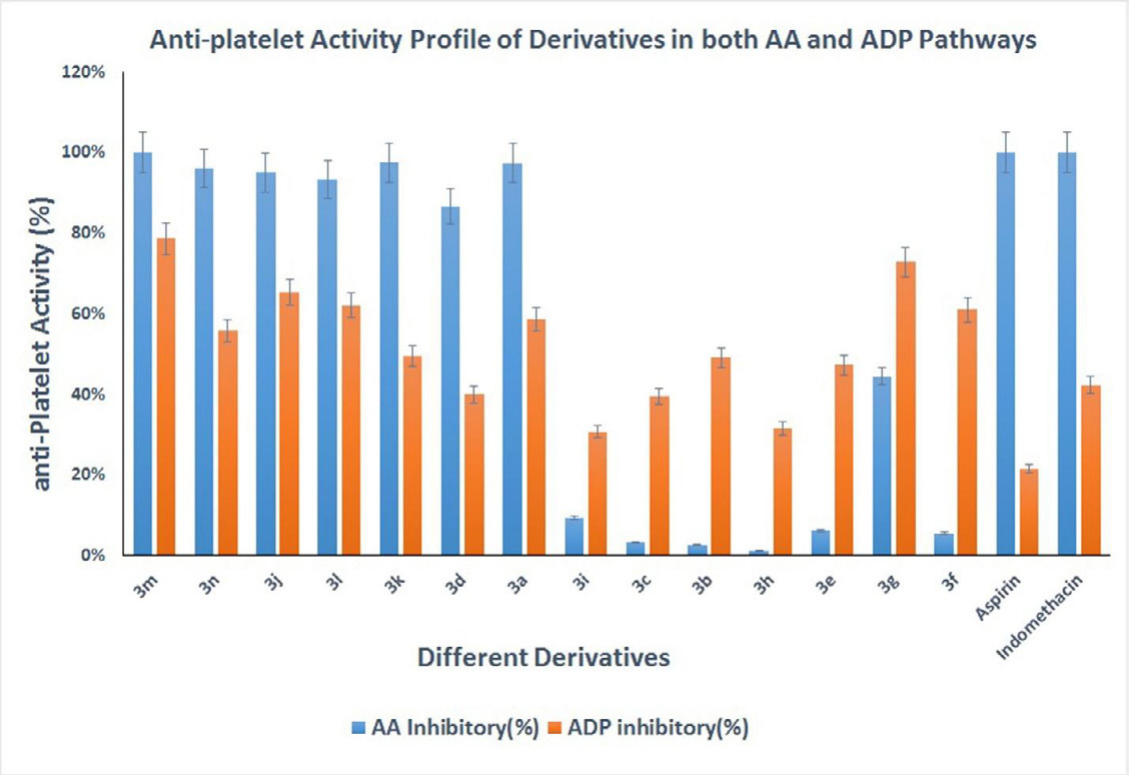
Antiplatelet activities of synthesized derivatives in both AA and ADP pathways at 1 mM concentration

Antiplatelet activities of all derivatives are provided in Table 1 and Figure 2.

The antiplatelet activities of the synthesized derivatives were evaluated using AA and ADP as platelet aggregation inducers (Table 1) based on Born’s procedure, as described in the method section (15). All the derivatives were initially tested at one mM, and IC_50_ was measured for the derivatives that inhibit platelet aggregation by more than 50% in the concentration of 1 mM.

All the prepared derivatives inhibited platelet aggregation induced by ADP between 30% and 80% at 1 mM concentration, despite their structural difference. Compound *3m* and *3g* with IC_50 _values of 401 µM and 553 µM were the most potent inhibitors against ADP, respectively. Although, all of the synthesized derivatives showed lower potency than indomethacin. As Figure 2 shows, the electron-withdrawing or releasing nature of the substituent on the phenyl ring did not significantly affect the antiplatelet potency, as the position of substitution does not have any valuable effect in the same manner on the ADP pathway. These results also imply that the phenyl ring possibly does not play a significant role as a pharmacophoric group and hydrazone moiety is the essential pharmacophore. Among the derivatives, compound *3m* (IC_50 _= 117 µM), *3n* (IC_50 _= 268 µM), and *3k* (IC_50 _= 302 µM) exhibited higher activities against platelet aggregation induced by AA. Whereas compounds *3h*, *3b*, *3c*, and 3f were inactive. It seems that the derivatives with electron-releasing substituent (hydroxyl, methoxy, and methyl group) have better inhibition activity against the aggregation induced by AA. Other studies support these findings, as Tehrani *et al.* have stated that the methoxy group increases antiplatelet activity in the related hydrazone-based structures (12). Substitution on phenyl ring by electron-withdrawing group on the other side is responsible for the significant decrease in their activities. It seems that the electron-rich aromatic rings connected to NH are the leading cause of antiplatelet activity in hydrazone-containing structures, as Mashayekhi *et al.* has reported earlier (11). Comparing compounds 3k and 3l exhibits that steric hindrance can cause a decrease in inhibitory activity against AA.

*Docking studies*

Based on these initial antiplatelet results, to complete and verify our results with computational modeling and to get more scientific logic for future designs, we ran molecular docking studies on the most active derivative (electron-rich one (*3m*), and the most electron-deficient derivative (*3g*) against AA, with their possible potential target cyclooxygenase-1 (COX-1).

**Figure 3:**
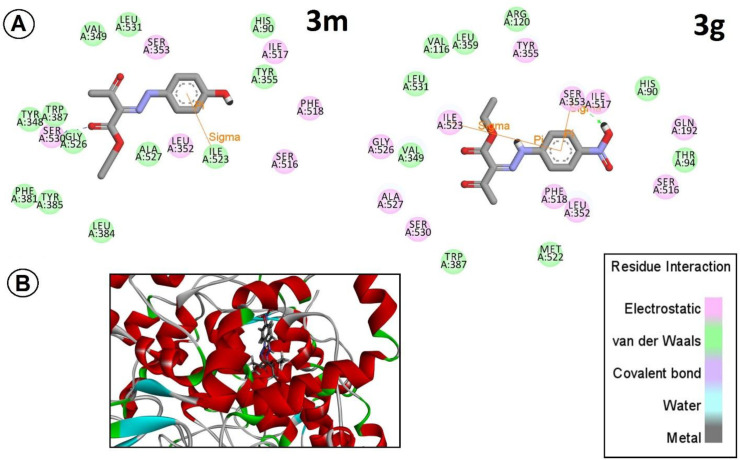
(A) Schematic diagram for interactions of *3m*, and *3g* with the COX-1 active site generated by Discovery studio V3.5; hydrogen bonds are showed as blue dashed lines. Residues involved in hydrophobic interactions with the ligands are shown as orange rays. (B) Interaction of compounds *3m* and *3g* with the COX-1 active site. The image was generated with PyMOL and Discovery studio V3.5

The best-docked pose with the lowest energy, calculated by GOLD Protein-Ligand Docking Software, was selected and analyzed with PyMOL and Discovery studio V3.5. The positions of compounds *3m* and *3g* in the binding site are shown in overlay mode in Figure 3A; furthermore, the residual interactions of both of *3m* and *3g* as representative of electron-rich and electron-deficient derivative with essential amino acids of the active site were clarified in Figure 3B. It was observed that 3m as the most potent derivative, has hydrogen bonding with Ser530 (in a distance of 1.87 Aº) as Amidi *et al.* stated in their recent paper (16), and another p-sigma interaction mainly with Ile 523 (in a distance of 2.34Aº). On the other hand, *3g* showed different interactions with surrounded amino acids (two p-s interactions in a distance of 2.51Aº with Ser353 and with Ile523 in a distance of 2.70Aº). This differential positioning maybe is the best explanation for different potencies as obtained in the experimental antiplatelet assessment. So based on initial docking studies, it seems that our chemical structures can obtain proper orientation within the active site of the enzyme, but this orientation differs in electron-rich and electron-deficient derivatives to some extent. In future studies, we hope to design some other derivatives to get more and more potent compounds.

**Table 1:** Antiplatelet aggregation activity of the synthesized Derivatives, both in AA and ADP protocols

Derivative	X	AA		ADP	
		Inhibition^a^ (%)	IC_50 _(µM)	Inhibition^a^ (%)	IC_50 _(µM)
***3a***	H	97.3	420	58.6	717
***3b***	4-Cl	2.6	-	49.1	-
***3c***	4-Br	3.1	-	39.3	-
***3d***	4-CH_3_	86.6	457	39.9	-
***3e***	3-NO_2_	6.2	-	47.2	-
***3f***	2-NO_2_	5.4	-	60.9	-
***3g***	4-NO_2_	44.4	-	72.9	553
***3h***	3,4-*di*Cl	1.1	-	31.5	-
***3i***	3-Cl	9.2	-	30.7	-
***3j***	2,6-*di*CH_3_	95	360	65.3	620
***3k***	2-CH_3_	97.5	302	49.5	-
***3l***	2,3-*di*CH_3_	93.3	346	62.1	678
***3m***	4-OH	100	117	78.6	401
***3n***	4-OCH3	96	268	55.8	-
**Indomethacin** ^b^		100	3	42.2	-
**Aspirin** ^b^		100	30	21.4	-

^a^The derivatives tested at 1 mM concentration.

^b^The standard tested at 250 µM concentration.

AA pathway potency trend: *3m* > *3n* > *3k* > *3l* > *3j* >*3a* > *3d*.

ADP pathway potency trend: *3m* > *3g* > *3j* > *3l* > *3a*.

## Conclusion

In this study, we have synthesized ethyl-2-(2-phenylhydrazono)-3-oxobutanoate derivatives and evaluated their antiplatelet activity against AA and ADP platelet inducers. The studied derivatives are more effective in inhibiting platelet aggregation induced by AA. Among the synthesized derivatives, compound ***3m*, **Ethyl-2-(2-(4-hydroxyphenyl) hydrazinylidene)-3-oxobutanoate, had the most inhibition effect against AA and ADP. Although the synthesized compounds exhibited lower potency compared with aspirin, these remarkable results encouraged us to further researches on these compounds in the future.
